# Globally distributed occurrences utilised in 200 spider species conservation profiles (Arachnida, Araneae)

**DOI:** 10.3897/BDJ.7.e33264

**Published:** 2019-04-02

**Authors:** Pedro Cardoso, Vaughn Shirey, Sini Seppälä, Sergio Henriques, Michael L Draney, Stefan Foord, Alastair T Gibbons, Luz A Gomez, Sarah Kariko, Jagoba Malumbres-Olarte, Marc Milne, Cor J Vink

**Affiliations:** 1 LIBRe - Laboratory for Integrative Biodiversity Research, Finnish Museum of Natural History, University of Helsinki, Helsinki, Finland LIBRe - Laboratory for Integrative Biodiversity Research, Finnish Museum of Natural History, University of Helsinki Helsinki Finland; 2 IUCN SSC Spider & Scorpion Specialist Group, Helsinki, Finland IUCN SSC Spider & Scorpion Specialist Group Helsinki Finland; 3 Georgetown University, Department of Biology, Washington, DC, United States of America Georgetown University, Department of Biology Washington, DC United States of America; 4 University College London, London, United Kingdom University College London London United Kingdom; 5 University of Wisconsin-Green Bay, Green Bay, United States of America University of Wisconsin-Green Bay Green Bay United States of America; 6 University of Venda, Thohoyandou, South Africa University of Venda Thohoyandou South Africa; 7 University of Nottingham, Nottingham, United Kingdom University of Nottingham Nottingham United Kingdom; 8 Universidad Nacional de Colombia, Bogotá, Colombia Universidad Nacional de Colombia Bogotá Colombia; 9 Museum of Comparative Zoology, Harvard University, Cambridge, United States of America Museum of Comparative Zoology, Harvard University Cambridge United States of America; 10 cE3c - Centre for Ecology, Evolution and Environmental Changes, University of the Azores, Angra do Heroísmo, Portugal cE3c - Centre for Ecology, Evolution and Environmental Changes, University of the Azores Angra do Heroísmo Portugal; 11 University of Indianapolis, Indianapolis, United States of America University of Indianapolis Indianapolis United States of America; 12 Canterbury Museum, Christchurch, New Zealand Canterbury Museum Christchurch New Zealand

**Keywords:** Arthropoda, bibliography search, IUCN, threat status

## Abstract

**Background:**

Data on 200 species of spiders were collected to assess the global threat status of the group worldwide. To supplement existing digital occurrence records from GBIF, a dataset of new occurrence records was compiled for all species using published literature or online sources, from which geographic coordinates were extracted or interpreted from locality description data.

**New information:**

A total of 5,104 occurrence records were obtained, of which 2,378 were from literature or online sources other than GBIF. Of these, 2,308 had coordinate data. Reporting years ranged from 1834 to 2017. Most records were from North America and Europe, with Brazil, China, India and Australia also well represented.

## Introduction

Spiders (Arachnida, Araneae) are a largely under-represented group amongst reported biodiversity occurrence records in the Global Biodiversity Information Facility (GBIF; Troudet et al. 2017). As such, aggregating new information regarding their distribution through time and space is crucial towards remedying shortfalls associated with the lack of data on species distributions – the Wallacean Shortfall (Lomolino 2004). These knowledge gaps can confound conservation efforts, particularly of invertebrates, a group that is already largely understudied (Cardoso et al. 2011).

A sample of 200 species of spiders were randomly selected from the World Spider Catalog (2018) as required by IUCN for the Sampled Red List Index. The World Spider Catalogue is an updated global database containing all recognised species names for the group and the best source of information for this type of analysis. Species data were collected from all taxonomic bibliography available at the World Spider Catalog 2018 and complemented by data in other publications found through Google Scholar or other sources (https://www.biodiversitylibrary.org; https://login.webofknowledge.com; http://srs.britishspiders.org.uk; http://symbiota4.acis.ufl.edu/scan/portal; https://lepus.unine.ch; http://www.tuite.nl/iwg/Araneae/SpiBenelux/?species; https://atlas.arages.de; https://arachnology.cz/rad/araneae-1.html; http://biodiversityresearch.org/research/biogeography/iberia).

These data were used previously in assessing the global threat status of spider species worldwide (Seppälä et al. 2018a, Seppälä et al. 2018b, Seppälä et al. 2018c, Seppälä et al. 2018d). This will serve as the basis for a future Sampled Red List Index (SRLI) for spiders. SRLI are typically employed to assess the conservation priorities and trends of large organismal groups and are thus suited for assessing the conservation trends of large taxa as a whole. The present paper compiles all data used in these assessments beyond those already present in GBIF and makes accessible all geographical information currently available on these 200 species.

## Geographic coverage

### Description

Global.

## Taxonomic coverage

### Taxa included

**Table:** 

Rank	Scientific Name	Common Name
order	Araneae	Spiders

## Temporal coverage

**Data range:** 1834-1-01 – 2017-12-31.

## Usage rights

### Use license

Creative Commons Public Domain Waiver (CC-Zero)

## Data resources

### Data package title

Global Spider Red List Index

### Resource link

http://ipt.pensoft.net/resource?r=srli_global_araneae

### Number of data sets

1

### Data set 1.

#### Data set name

SRLI_Global_Araneae

#### Number of columns

21

#### Description

The goal of this project is to serve as the basis for a future Sampled Red List Index (SRLI) for spiders.

**Data set 1.** 

Column label	Column description
occurrenceID	An identifier for the Occurrence (as opposed to a particular digital record of the occurrence).
basisOfRecord	The specific nature of the data record.
taxonRank	The taxonomic rank of the most specific name in the scientificName.
phylum	The full scientific name of the phylum or division in which the taxon is classified.
class	The full scientific name of the class in which the taxon is classified.
order	The full scientific name of the order in which the taxon is classified.
family	The full scientific name of the family in which the taxon is classified.
genus	The full scientific name of the genus in which the taxon is classified.
specificEpithet	The name of the first or species epithet of the scientificName.
scientificName	The full scientific name, with authorship and date information if known.
scientificNameAuthorship	The authorship information for the scientificName formatted according to the conventions of the applicable nomenclaturalCode.
verbatimLocality	The original textual description of the place.
country	The name of the country or major administrative unit in which the Location occurs.
decimalLatitude	The geographic latitude (in decimal degrees, using the spatial reference system given in geodeticDatum) of the geographic centre of a Location.
decimalLongitude	The geographic longitude (in decimal degrees, using the spatial reference system given in geodeticDatum) of the geographic centre of a Location.
geodeticDatum	The ellipsoid, geodetic datum or spatial reference system (SRS) upon which the geographic coordinates given in decimalLatitude and decimalLongitude as based.
georeferencedBy	A list (concatenated and separated) of names of people, groups or organisations who determined the georeference (spatial representation) for the Location.
georeferenceProtocol	A description or reference to the methods used to determine the spatial footprint, coordinates and uncertainties.
verbatimEventDate	The verbatim original representation of the date and time information for an Event. The nature of the event is dependent on the source, including individual samples or entire sampling seasons in single sites or regions.
eventDate	The date-time or interval during which an Event occurred.
associatedReferences	A list (concatenated and separated) of identifiers (publication, bibliographic reference, global unique identifier, URI) of literature associated with the Occurrence.

## Additional information

**Figure 1.**
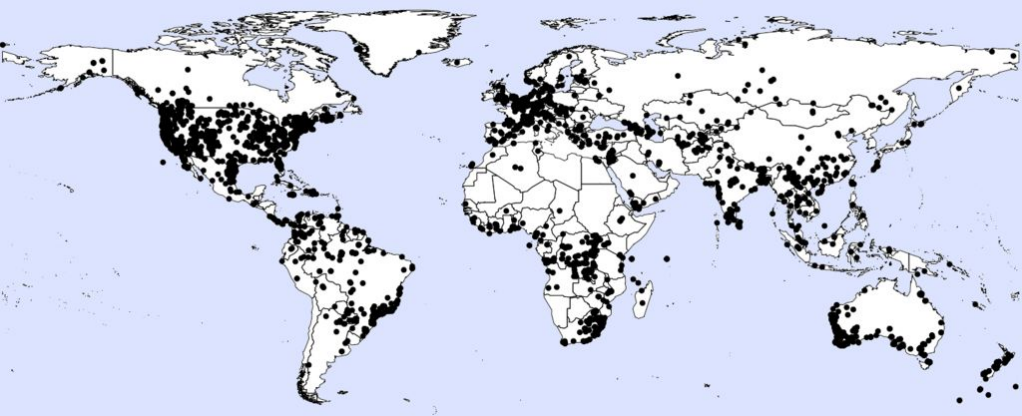
Map of distribution of records.

**Figure 2.**
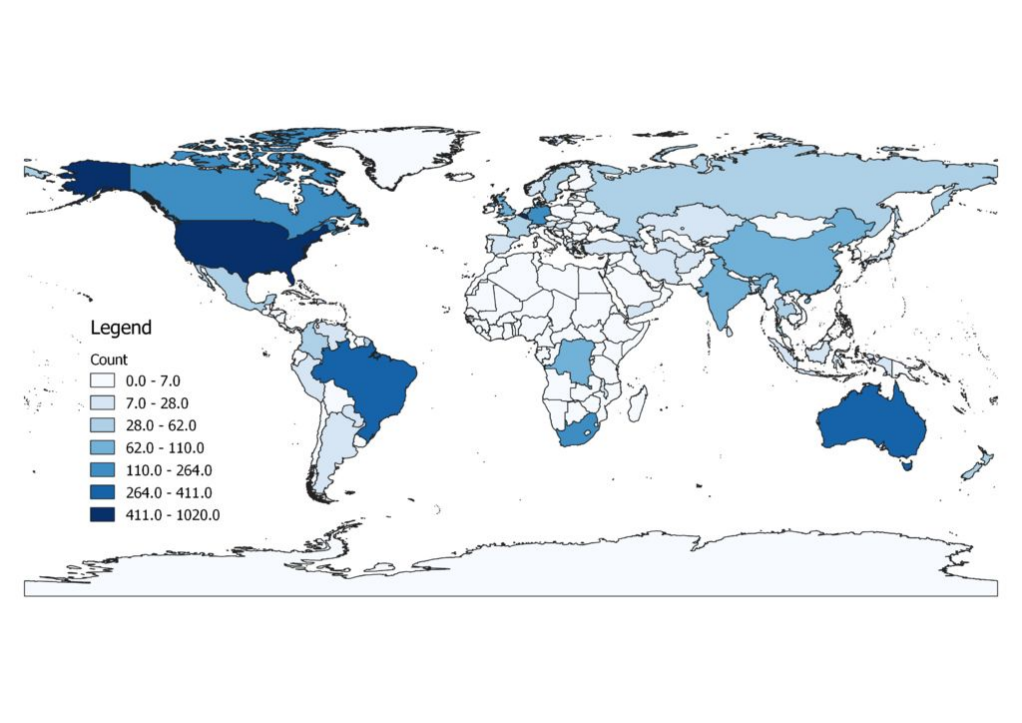
Map of records per country.

**Figure 3.**
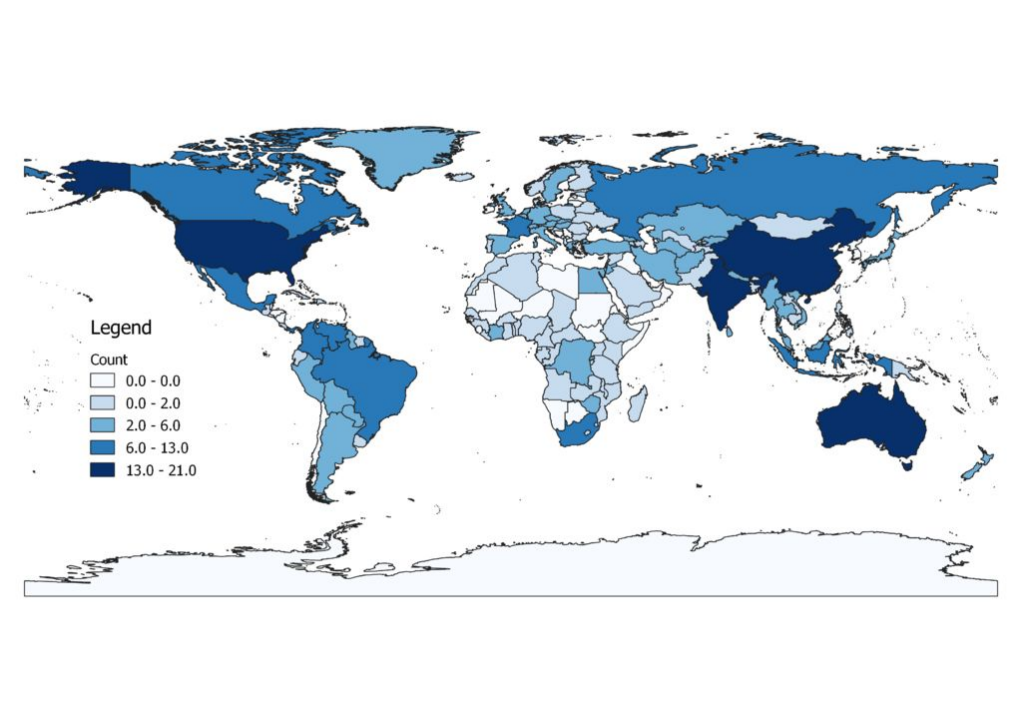
Map of species per country.

**Figure 4.**
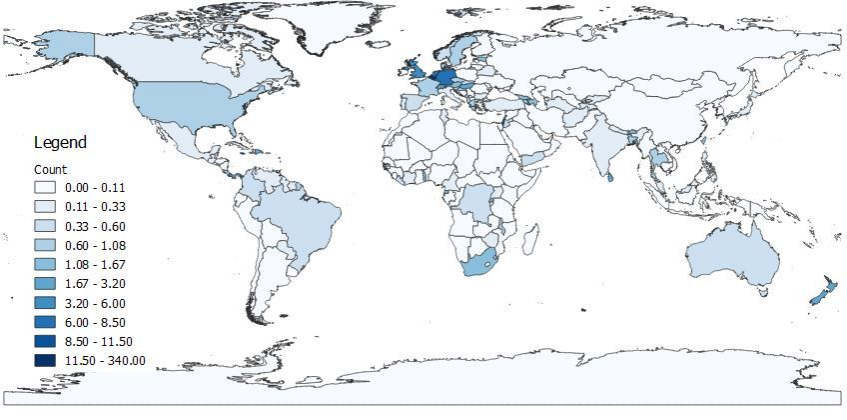
Number of records per country, standardised by country area.

**Figure 5.**
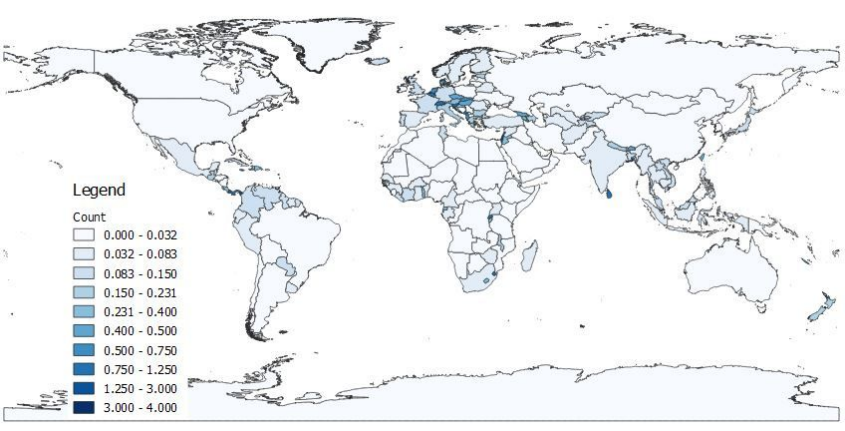
Number of species per country, standardised by country area.

A total of 5,104 occurrence records were obtained, of which 2,378 were from literature or online sources other than GBIF and are included in this dataset. Of these, 2,308 had coordinate data. We should note that, following the IUCN guidelines, records outside the native range of a species are not included in analyses, here or in the conservation profiles. Reporting years ranged from 1834 to 2017. Most records of the 200 species that we selected randomly from all those known at the global level were from a few better-known regions (Fig. 1). Higher numbers of records were found in the USA, Canada, Brazil and Australia (Fig. 2) and higher numbers of species in the USA, China, India and Australia (Fig. 3). Yet, when corrected by area, higher densities of both records and species were found in several European countries (Figs 4, 5).

**Figure 6.**
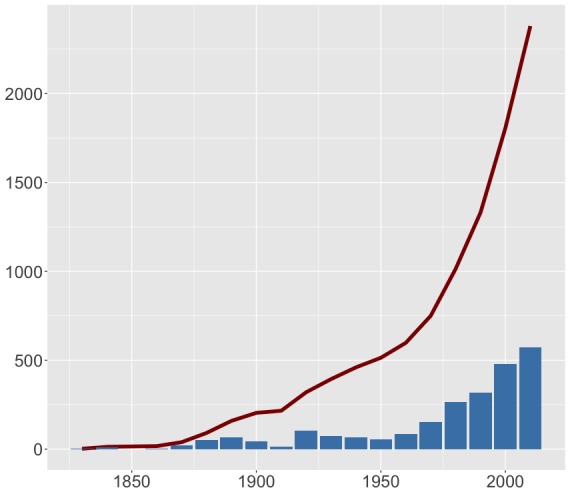
Number of records published per decade (bars) and respective accumulation curve (line).

**Figure 7.**
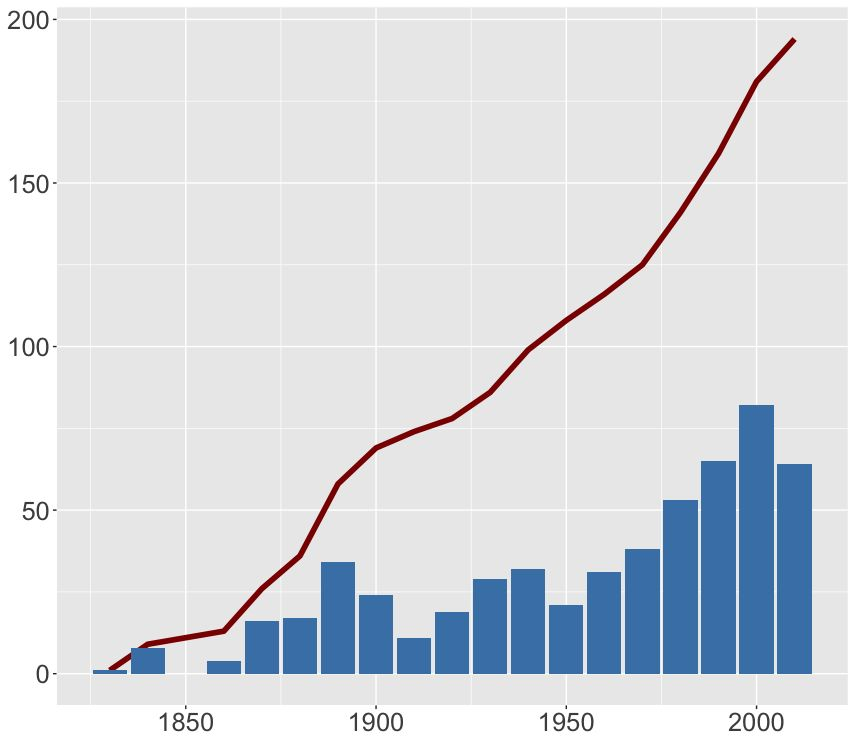
Number of species published per decade (bars) and respective accumulation curve (line).

We also assessed temporal trends within the data. As is common for multiple taxa and regions, the number of records increased with time, with most being published during the last few decades (Fig. 6). The number of unique species recorded per decade is also increasing, although in a less dramatic way (Fig. 7).

**Figure 8.**
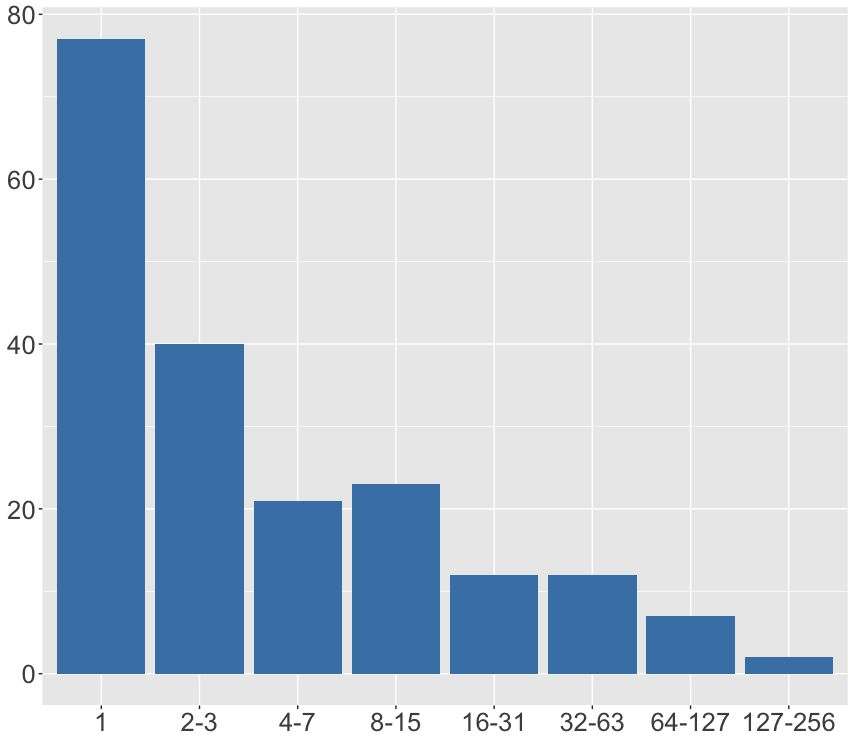
Abundance distribution of all species records.

Finally, the species (record) abundance distribution (Fig. 8) shows that most species have very few records, with more than one third of the species having a single record and more than half with three or less.

Although we have only looked at a sample of 200 species, given the random nature of their selection, the trends we found should be representative of spiders as a whole. There is a clear geographical bias of available data towards some regions, an increase in the number of studies reporting useful locality data during the latter decades and yet, most species at a global level are still almost entirely unknown beyond a name and an often old and incomplete taxonomic description.
